# Exercise and physical activity reference for health promotion 2006 (EPAR2006)

**DOI:** 10.2188/jea.17.177

**Published:** 2007-09-01

Dear Sir:

We would like to introduce the "Exercise and Physical Activity Reference for Health Promotion 2006" published by the Office for Lifestyle-Related Diseases Control, General Affairs Division, Health Service Bureau, Ministry of Health, Labour and Welfare of Japan, to the members of the Japan Epidemiological Association. Because we are the members who produced this report, we hope to familiarize association members with it, and move ahead with many outstanding studies on the relationship between physical activity or fitness and lifestyle-related diseases in future revisions.

The report was first established as "the Recommended Exercise Allowances for Health Promotion" in 1989 to prevent coronary heart disease. It is now more than 15 years since the former report, and with rapid aging of the population, Japan's morbidity pattern has changed. Lifestyle-related diseases such as diabetes, hypertension and hyperlipidemia have come to the fore. Furthermore, the disease concept and diagnostic standards for "metabolic syndrome" were explicated at 8 related academic societies in April 2005.

In 2000, a 10-year strategy for "National Health Promotion in the 21st Century (Health Japan 21)" was established. The goal for the proportion of those who regularly exercise was put at 39% for men and 35% for women. However, mid-term evaluation failed to increase the proportion. Under these circumstances, it was decided to draw up recommendations for exercise based on the latest scientific findings, designed to maintain and promote the health of people and prevent lifestyle-related diseases through improving their habits of physical activity and exercise.

A systematic review was conducted to determine the reference quantity of physical activity, exercise, and fitness level for prevention of lifestyle-related diseases. The English and Japanese literature published before April 11, 2005 was searched using PubMed and Japana Centra Rebuo Medicina. First, 8,134 articles were selected from these databases. Following the primary screening by examining their titles and abstracts, and then intensive reading of the full texts, 84 excellent articles were finally selected.

In "Exercise and physical activity reference for health promotion 2006 (EPAR2006)", the reference values were set as 23 METs hour/week for physical activity and 4 METs hour/week (2 to 10 METs hour/week) for exercise. Approximately 60 minutes per day at an intensity of 3 METs is equivalent to the physical activity reference value. If activity were composed mainly of walking, the quantity of physical activity would be equivalent to 8,000 to 10,000 steps per day. As for exercise, 60 minutes of walking or 35 minutes of jogging or playing tennis are equivalent to the reference value. In addition, the reference values were shown for the maximal oxygen uptake for health promotion by gender and age.

EPAR2006 will be updated periodically according to new scientific findings. Now the following issues are considered necessary for future research.

1. Accumulation of scientific evidence on physical activity, exercise and physical fitness of Japanese.
2. Standardization of methods to assess physical activity.
3. Evaluation of physical activity, exercise and physical fitness according to gender and age group.
4. Evaluation of specific indices for muscle strength and muscle volume.
5. Evaluation of the upper limit of physical activity and exercise for health promotion.
6. Determination of the effect of medical expenses saved by pursuing the reference levels of physical activity and exercise.

To promote this report among the population at large and to achieve the reference values, the "Exercise Guideline 2006" was also established in 2006. The full text of both the EPAR2006 and "Exercise Guideline 2006" are provided by PDF files on the Ministry of Health, Labour and Welfare of Japan Website (http://www.mhlw.go.jp/bunya/kenkou/undou.html) in the Japanese version, and the National Institute of Health and Nutrition, Japan (http://www.nih.go.jp/eiken/programs/eiyo_eiyo.html) in the Japanese and English version (English version is EPAR2006 only). The English version for the "Exercise Guideline 2006" and Chinese and Korean version for EPAR2006 will be provided on our institute website soon.

Kazuko Ishikawa-Takata, Izumi Tabata (Program of Health Promotion and Exercise, National Institute of Health and Nutrition, Japan)

